# Robust immunohistochemical detection of α-synuclein, tau and amyloid-β in human brain tissue archived for up to 78 years

**DOI:** 10.17879/freeneuropathology-2026-9375

**Published:** 2026-03-18

**Authors:** Mie Kristine Just, Kristina Bang Christensen, Martin Wirenfeldt, Torben Steiniche, Laura Parkkinen, Liisa Myllykangas, Per Borghammer

**Affiliations:** 1 Department of Clinical Medicine, Aarhus University, Aarhus, Denmark; 2 Lundbeck Foundation Parkinson’s Disease Research Center (PACE), Aarhus University Hospital, Aarhus, Denmark; 3 Department of Nuclear Medicine, Aarhus University Hospital, Aarhus, Denmark; 4 Department of Pathology, Aarhus University Hospital, Aarhus, Denmark; 5 Department of Pathology, University Hospital of Southern Denmark, Esbjerg, Denmark; 6 Department of Regional Health Research and BRIDGE (Brain Research – Inter Disciplinary Guided Excellence), University of Southern Denmark, Odense, Denmark; 7 The Oxford Brain Bank, Nuffield Department of Clinical Neurosciences, University of Oxford, Oxford, United Kingdom; 8 Department of Pathology, University of Helsinki, Helsinki, Finland; 9 Department of Pathology, HUS Diagnostic Center, Helsinki University Hospital, Helsinki, Finland

**Keywords:** Alpha-synuclein, Amyloid-beta, Antigen retrieval, Archival tissue, Hyperphosphorylated tau

## Abstract

**Objective**: Brain branks preserve extensive material relevant to neurodegenerative disease research. As these collections age, tissue becomes archival, raising the question of whether long-term fixed and stored human brain tissue remains suitable for contemporary immunohistochemical analyses.

**Materials and Methods**: Forty-one autopsy brains collected between 1946 to 1980 were examined. For each case, midbrain and hippocampus were available both as original paraffin-embedded blocks and as tissue stored long term in fixative. New paraffin blocks were prepared from the long-term fixated tissue. Sections from original and newly prepared blocks were immunohistochemically stained for α-synuclein, hyperphosphorylated tau and amyloid-β. Immunoreactivity was assessed using semi-quantitative scoring.

**Results**: Original blocks consistently showed good staining intensity and morphological preservation for each protein pathology. Newly prepared blocks showed slightly lower semi-quantitative scores for Lewy-related pathology, without statistically significant differences, except for astrocytic α-synuclein in the substantia nigra in cases from the 1960s. Tau pathology displayed modestly reduced labelling, particularly of the neuropil threads and neurofibrillary tangles, most evident in cases from the 1950s. Amyloid-β-positive senile plaques showed similar or slightly higher scores in newly prepared blocks, with no significant differences across regions.

**Conclusion**: Human brain tissue preserved as paraffin-embedded blocks or stored in fixative for up to 78 years remains suitable for immunohistochemical analyses. Adequate-to-good detection of aggregated α-synuclein, hyperphosphorylated tau and amyloid-β is achievable, indicating preserved pathological hallmarks of Lewy Body Disease and Alzheimer’s Disease in archival tissue.

## Abbreviations

**Aβ** - amyloid-beta, **AD** - Alzheimer’s Disease, **αSyn** - alpha-synuclein, **CAA** – cerebral amyloid angiopathy, **Ent Cx** - entorhinal cortex, **FA** - formic acid, **FFPE** - formalin-fixed paraffin-embedded, **IHC** - immunohistochemical, **LB** - Lewy body, **LBD** - Lewy Body Disease, **LN** - Lewy neurite, **NAC** – non-amyloid-beta component of Alzheimer’s disease amyloid, **NFT** - neurofibrillary tangle, **NT** - neuropil thread, **PAG** - periaqueductal grey, **PD** - Parkinson’s Disease, **ROI** - region of interest, **SN** - substantia nigra, **SP** - senile plaque, **TO Cx** - temporo-occipital cortex

## Introduction

Alzheimer’s Disease (AD) and Lewy Body Disease (LBD), including Parkinson’s Disease (PD), are the most prevalent neurodegenerative diseases, affecting millions of people worldwide. Neuropathological assessment is primarily based on histological and immunohistochemical (IHC) stains of paraffin-embedded brain tissue. In particular, IHC staining of paraffin-embedded tissue is an invaluable technique used for both diagnostic and research purposes. The pathology of LBD and AD is characterised by dense protein aggregates including intracellular accumulation of α-synuclein (αSyn) into Lewy bodies (LBs) and Lewy neurites (LNs); hyperphosphorylated tau assembling into neurofibrillary tangles (NFTs) and neuropil threads (NTs), and the extracellular accumulation of amyloid-β (Aβ) into senile plaques (SPs).

Staging systems of AD and LBD are based on neuropathological assessment of brain bank material. Systematic brain banking began in the 1940s–1950s [1]. Many early brain collections originated from routine hospital autopsies, whereas modern collections often come from donors enrolled in specific brain donation programs. In the 1950s, autopsy rates began to decline steadily, decreasing from 41.1 % in 1964 to less than 5 % in 1995 [2]. The decline in autopsy rates, and consequently reduced input to brain banks, highlights the importance of assessing and re-assessing archived tissue already available.

In addition to storing formalin-fixed paraffin-embedded (FFPE) blocks, many brain banks and departments of anatomy and pathology also preserve tissue in formalin or similar fixatives. Formalin fixation involves rapid penetration of formaldehyde into the tissue, formation of covalent bonds and cross-linking, i.e. the formation of methylene bridges, with complete fixation typically obtained within 24–48 hours [3]. However, fixation is a continuous process, and prolonged fixation may result in reduced antigenicity and IHC efficacy due to structural alterations of epitopes [6] and decreased accessibility of antigens. Furthermore, over longer periods, formalin may oxidize into formic acid (FA), further diminishing antigenicity and compromising IHC results [7].

Common methods for unmasking antigens, i.e. antigen retrieval, include enzymatic digestion (e.g. proteinase K) or heating in citric acid or FA pretreatment. Some studies have shown that prolonged fixation of brain tissue can compromise IHC staining for αSyn, Aβ and tau, depending on the antibodies used [8]. One study investigated prolonged fixation for up to 14 years, and reported reduced antigenicity for αSyn, Aβ, tau, ubiquitin and p62. However, with appropriate combination of antibody and antigen retrieval, antigenicity could be restored for some targets [11].

Antigen preservation and recovery in archival FFPE tissue blocks from 1960 to 2010 have also been investigated. These studies found that most antigens remain well-preserved over several decades. Cytoplasmic antigens were maintained for 60 years or more, while nuclear and membranous antigens were more prone to decay. The study suggested that deeper sectioning and modified pretreatment can optimise antigenicity in old archival FFPE blocks [12].

The Danish Brain Collection contains nearly 10,000 brains from patients admitted to psychiatric hospitals in Denmark, collected between 1945 and 1982 under the Institute of Brain Pathology, Psychiatric Hospital Risskov, Denmark. Approximately 5,500 patients had a diagnosis of dementia, and 1,500 had a diagnosis of schizophrenia. Other types of disorders include manic-depressive disorder, depression, PD, brain tumours, stroke, and more.

The aim of the present study was to evaluate whether very old archival brain material, i.e. both original paraffin-embedded blocks as well as newly sampled tissue with fixation times up to 78 years, remain viable for IHC detection of protein aggregates associated with LBD and AD. The extent of immunoreactive structures were assessed using 41 cases positive for LBD and/or AD protein pathologies, sampled across the decades covered by the Danish Brain Collection.

## Materials and Methods

### Tissue samples

This study was conducted on brain tissue samples obtained from 41 postmortem brains from which the midbrain and the hippocampus were available both as original paraffin-embedded blocks and in fixative for each case. All included brains displayed Lewy pathology and/or AD pathology. We examined brains that were collected between 1946 and 1980 as part of the brain banking period of the Danish Brain Collection (University of Southern Denmark, Odense). Ethical approval (1-10-72-200-20) was obtained from the Scientific Ethics Committees for the Central Denmark Region.

A minimum of 10 cases were selected from the last half of each decade, i.e. from 1945–1949, 1955–1959, 1965–1969, and 1975–1980. Two cases from the 1940s group were excluded; one due to unusable fixated tissue and one because sections from the original block could not adhere to the slides.

The original blocks had been processed following standard procedures at the time of autopsy and neuropathological assessment and were stored in cardboard boxes at room temperature. To ensure compatibility with our microtome, these original samples were re-embedded in fresh paraffin. The re-embedding procedure involved melting the paraffin of the original block on a warm plate, replacing it with new paraffin in either regular or "mega" moulds depending on the tissue size, and finally mounting new cassettes compatible with the microtome.

Available information on fixative, concentrations and fixation duration was variable. The type of fixative was available in 24 of 43 cases, i.e. 23 brains were formalin-fixed, and one brain was fixed in Lilli’s acetate-formol. For the remaining 19 cases, the type of fixative was unknown, though most were presumably fixed in formalin. The duration of fixation of the original blocks is unknown, but standard procedures of the time were followed. Long-fixated brains were stored in fixative in plastic containers at room temperature. Original blocks and fixed brains were stored together in basement storage facilities at the Psychiatric Hospital Risskov, Aarhus, Denmark from sampling to 2018 and at University of Southern Denmark, Odense, Denmark from 2018 to present.

New blocks were sampled from the long-fixated brains, which had previously been cut into approximately 1 cm slices during the original neuropathological evaluation. The new blocks were sampled as close as possible to the original sampling site. Prolonged fixation times of the new blocks were calculated in years from the time of autopsy or death to the year of the new sampling. All tissue samples for this study were sampled between 2020 and 2024. Fixation times of the new blocks and storage times of the original blocks are summarised in **Table 1**.

**Table 1:** Demographics and tissue storage times

Decade	Case no.	Age at death	Sex	Clinical diagnosis	Postmortem delay, hours	Fixative	Storage time of original blocks, years	Fixation time of new blocks, years
1940s	1	72	M	Dementia + PD	N/A	Formalin	78	78
	2	64	M	Dementia	N/A	Formalin	77	77
	3	58	F	PD	26	Formalin	78	76
	4	90	F	Dementia	24	Formalin	76	76
	5	74	F	Dementia	19	Formalin	77	75
	6	76	M	Dementia	6	Formalin	77	75
	7	82	M	Dementia	18	Formalin	75	75
	8	79	F	Dementia	22	Formalin	75	74
	**9**	**77**	**F**	**Dementia**	**8**	**Formalin**	**74**	**N/A**
	**10**	**65**	**M**	**Dementia + PD**	**10**	**Formalin**	**78**	**N/A**
1950s	11	66	M	Dementia	39*	N/A	69	69
	12	86	M	Dementia + parkinsonism	7	Formalin	69	69
	13	73	F	PD	25	Formalin	69	67
	14	70	F	Dementia + PD	52	Formalin	66	66
	15	88	M	Dementia	28	Formalin	66	66
	16	73	M	Dementia	10	N/A	66	66
	17	77	M	Dementia + parkinsonism	14	Formalin	66	66
	18	75	M	PD	N/A	N/A	66	66
	19	73	F	Dementia + parkinsonism	N/A	N/A	65	65
	20	79	M	Dementia	113	Formalin	65	65
	21	70	M	Dementia	6	N/A	66	64
1960s	22	74	M	Dementia + parkinsonism	N/A	N/A	59	59
	23	67	M	Dementia	7	N/A	57	57
	24	50	M	Dementia	48*	N/A	57	57
	25	77	F	Dementia	13	Formalin	56	56
	26	62	F	Dementia + PD	16	Formalin	56	56
	27	69	M	PD	24*	N/A	57	55
	28	73	F	Dementia + PD	24	Formalin	55	55
	29	82	F	Dementia + PD	N/A	Formalin	55	55
	30	79	M	PD	48*	N/A	55	55
	31	68	F	Dementia + parkinsonism	24*	N/A	56	54
	32	86	F	Dementia	18	Formalin	56	54
	33	72	M	Dementia	N/A	N/A	55	53
1970s	34	75	M	Dementia	20*	N/A	49	49
	35	86	M	Dementia	24*	Formalin	49	47
	36	78	F	Dementia	N/A	N/A	48	46
	37	72	F	Dementia + parkinsonism	N/A	N/A	46	42
	38	80	F	Manic-depressive psychosis	48*	Lilli’s Acetate-formol	44	42
	39	75	F	Dementia	N/A	N/A	45	41
	40	72	F	Dementia	22*	Formalin	45	41
	41	77	F	Dementia + PD	N/A	N/A	45	41
	42	77	M	Dementia + PD	N/A	N/A	44	40
	43	72	M	Dementia + PD	N/A	N/A	44	40
		Avg. age	% M		Avg. PMD		Avg. storage time	Avg. fixation time
1940s	*n = 8*	*74.4*	*50*		*16.6*		*76.6*	*75.8*
1950s	*n = 11*	*75.5*	*80*		*32.7*		*66.6*	*66.3*
1960s	*n =12*	*71.6*	*60*		*24.7*		*56.2*	*55.5*
1970s	*n = 10*	*76.4*	*40*		*28.5*		*45.9*	*42.9*

*PMD is an approximation as only the date of autopsy is known. Abbreviations: **avg.** - average; **N/A** - not available; **PD** - Parkinson’s Disease; **PMD** - postmortem delay.

Based on the minimum recommended brain regions to be sampled by Montine and colleagues [13] and the regions originally sampled, the following areas were evaluated: the midbrain including the substantia nigra (SN) and periaqueductal grey (PAG), and the hippocampal block, including CA1 and CA2 of the hippocampus, the entorhinal cortex (Ent Cx) and the temporo-occipital cortex (TO Cx). Details on which regions were analysed for specific types of pathology are provided in **Table 2**.

**Table 2:** Regions analysed for each type of pathology

Pathology	Midbrain	Hippocampus	Cortex
Amyloid plaques	PAG	CA1 and CA2	Ent Cx + TO Cx
Cerebral amyloid angiopathy			Ent Cx + TO Cx
NFTs		CA1 and CA2	Ent Cx + TO Cx
NTs		CA1 and CA2	Ent Cx + TO Cx
LBs (LB-like inclusions*)	SN	CA2	Ent Cx + TO Cx
LNs	SN	CA2	Ent Cx + TO Cx
Astrocytic Syn	SN	CA2	Ent Cx + TO Cx

*Intracellular, intraneuritic and extracellular inclusions. Abbreviations: **CA1** - cornu ammonis 1; **CA2** - cornu ammonis 2; **Ent Cx** - entorhinal cortex; **PAG** - periaqueductal grey; **SN** - substantia nigra; **TO Cx** - temporo-occipital cortex.

### Immunohistochemistry

Immunohistochemical staining was performed with an automated Ventana Benchmark Ultra immunostainer (Ventana Medical Systems, Arizona, USA) to standardise the technique. Tissue sections of 4 μm were deparaffinized and subjected to antigen retrieval using Envision Flex Target Retrieval Solution (DAKO, Glostrup, Denmark) in a pressure cooker (decloaking chamber™ NxGen Manual, Biocare Medical, California, USA) at 95℃, followed by incubation in 98 % formic acid (VWR, Avantor, Pennsylvania, USA). Details on antigen retrieval are provided in **Table 3**. The remaining immunohistochemical staining steps were performed on the Ventana Benchmark Ultra (Ventana Medical Systems, Arizona, USA). Based on previous IHC studies on archival brain tissue, the 5G4 antibody was chosen for detecting αSyn as it had shown promising IHC results on long-fixed brain tissue. Sections were stained for αSyn (5G4 #847-0102004001, 1:5000, AJ Roboscreen GmbH Leipzig, Germany), Aβ (4G8 #9220-02, 1:14,000, BioLegend, California, USA) and hyperphosphorylated tau (AT8 #90206, 1:1000, Fujirebio, Pennsylvania, USA). Visualisation was achieved using Optiview DAB (Roche Diagnostics, Ventana, Arizona, USA), and haematoxylin (Roche Diagnostics, Ventana, Arizona, USA) was used for counterstaining.

**Table 3:** Immunohistochemistry: antibodies and antigen retrieval

Antibody	Clone	Vendor	Dilution	Target retrieval	Formic acid
αSyn	5G4	Roboscreen	1:5000	30 min, 95 °C, pH = 6	30 min
HP-Tau	AT8	Fujirebio	1:1000	20 min, 95 °C, pH = 9	5 min
Aβ	4G8	BioLegend	1:14,000	20 min, 95 °C, pH = 9	5 min

Abbreviations: **αSyn** - alpha-synuclein; **Aβ** - amyloid-beta; **HP-Tau** - hyperphosphorylated tau.

### Digital assessment

Whole slide scans of sections from the midbrain and hippocampal blocks including Ent Cx and TO Cx were captured on a digital slide scanner (S60, Hamamatsu, Hamamatsu City, Japan). Circular regions of interest (ROIs) of 2 mm diameter were assessed digitally at x20 magnification. The ROIs were placed at the hotspot of pathology within the assessed region.

### Assessment of pathology

All samples were analysed by the same evaluator. The extent of αSyn, tau and Aβ pathologies were semi-quantitatively assessed at a magnification of x20 as previously described [11]. In short, intracellular inclusions (LBs and NFTs) and extracellular aggregates (SPs) were scored: 0, no lesions; 1, 1 to 5 lesions; 2, 6 to 20 lesions; 3, more than 20 lesions. The neuritic densities of LNs and NTs and astrocytic density of αSyn were assessed on a semi-quantitative scale of 0–3: 0, absent; 1, occasional; 2, moderate; 3, severe. Briefly, occasionally present meant LNs/NTs/astrocytic αSyn had to be sought out. Densities were scored moderate, when they were readily seen. Lastly, densities were rated severe when numerous LNs/NTs/αSyn-positive astrocytes were present. The presence or absence of cerebral amyloid angiopathy (CAA) was noted.

Staining quality was estimated on a 3-step scale (good, acceptable, or poor). Briefly, the staining quality was assessed as good when the pathological structures were clearly labelled with no or very faint background stain. The staining was acceptable when structures were partially labelled but still detectable or were well-labelled but showed some background staining. The staining was assessed as poor if labelling was faint or the region showed excessive background staining. **Table 4** summarises the assessment of staining quality.

**Table 4: Staining Quality of Immunohistochemical Stains:**
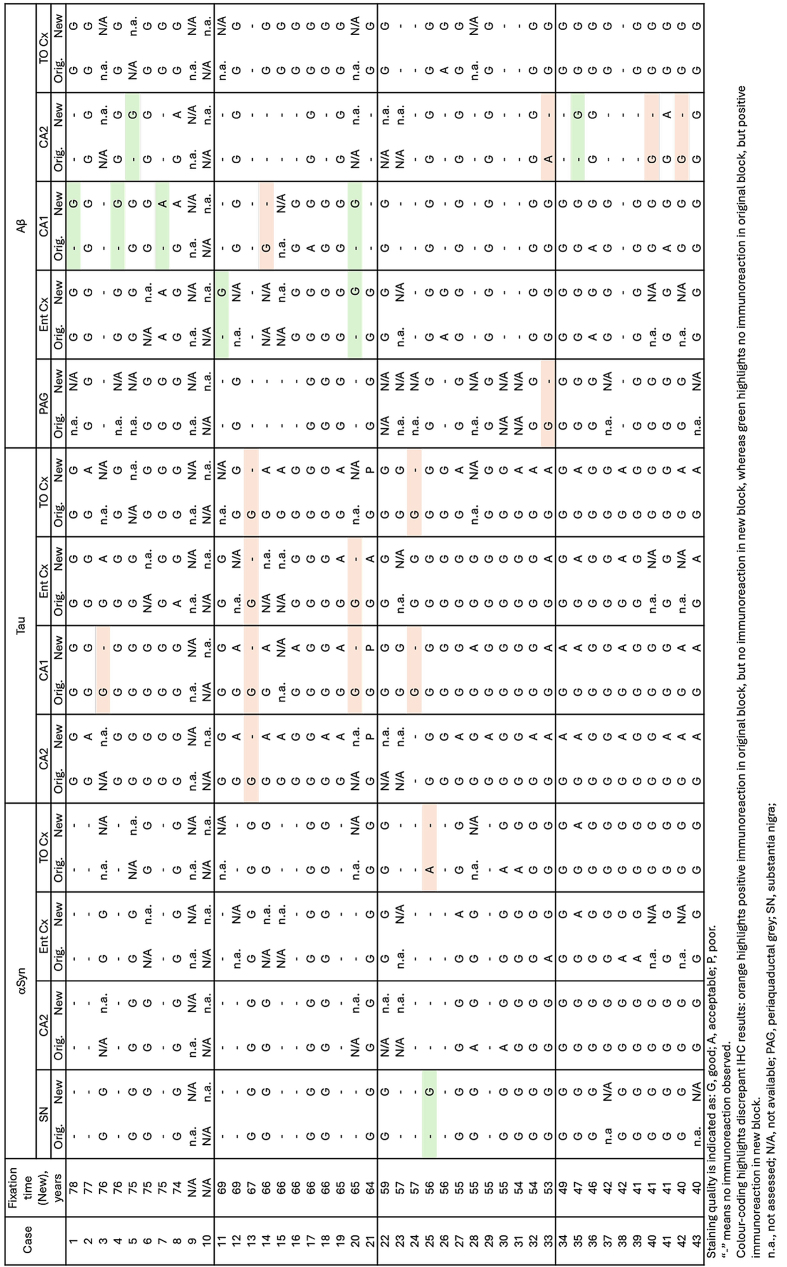


### Statistical Analysis

The statistical analyses were performed using GraphPad Prism version 10.6.1 for macOS, GraphPad Software, Boston, Massachusetts USA. The comparison of semi-quantitative assessments of the two types of preserved tissue was estimated applying the nonparametric Wilcoxon rank-sum test. P values are shown in tables by asterisks: *, p < 0.05; **, p < 0.01.

## Results

**Figure 1:**
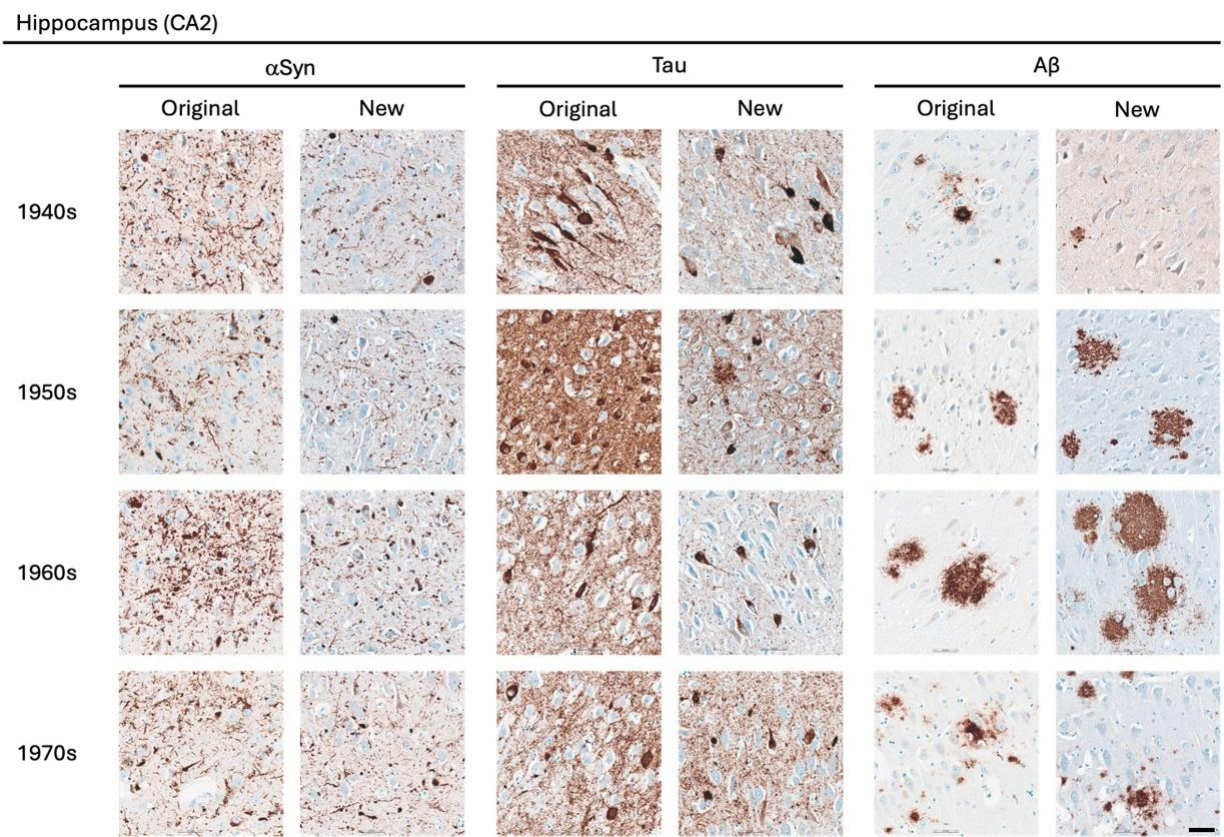
Representative images of immunohistochemical stains of CA2 of the hippocampus for originally paraffin-embedded blocks (Original) and for newly paraffin-embedded blocks upon prolonged fixation (New). **αSyn** Photomicrographs comparing 5G4 IHC staining seen in same case per decade. **Tau** Photomicrographs comparing AT8 IHC staining seen in same case per decade. Note that intense staining represents specific tau staining. **Aβ** Photomicrographs comparing 4G8 IHC staining seen in same case per decade. Scale bar = 50 μm. Abbreviations: αSyn, alpha-synuclein; Aβ, amyloid-beta.

**Figure 2:**
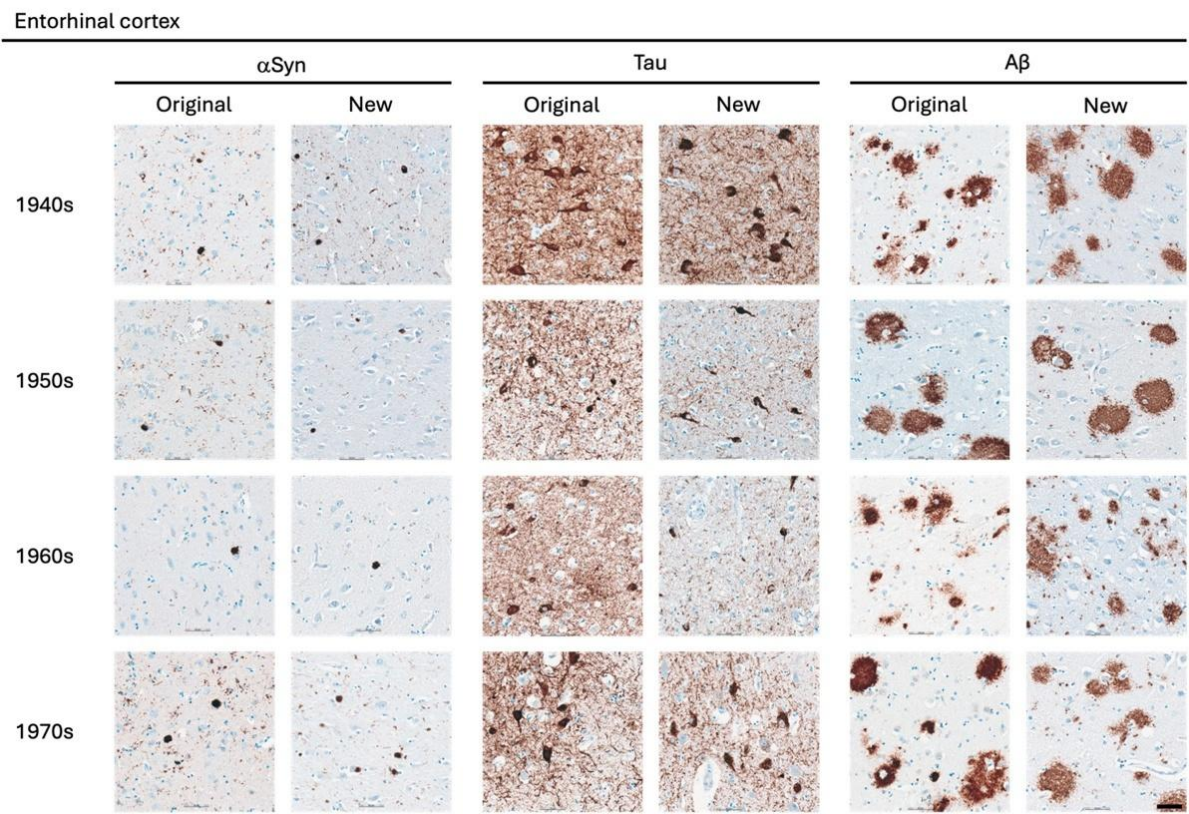
Representative images of immunohistochemical stains of entorhinal cortex for originally paraffin-embedded blocks (Original) and for newly paraffin-embedded blocks upon prolonged fixation (New). **αSyn** Photomicrographs comparing 5G4 IHC staining seen in same case per decade. **Tau** Photomicrographs comparing AT8 IHC staining seen in same case per decade. **Aβ** Photomicrographs comparing 4G8 IHC staining seen in same case per decade. Scale bar = 50 μm. Abbreviations: αSyn, alpha-synuclein; Aβ, amyloid-beta.

**Figure 3:**
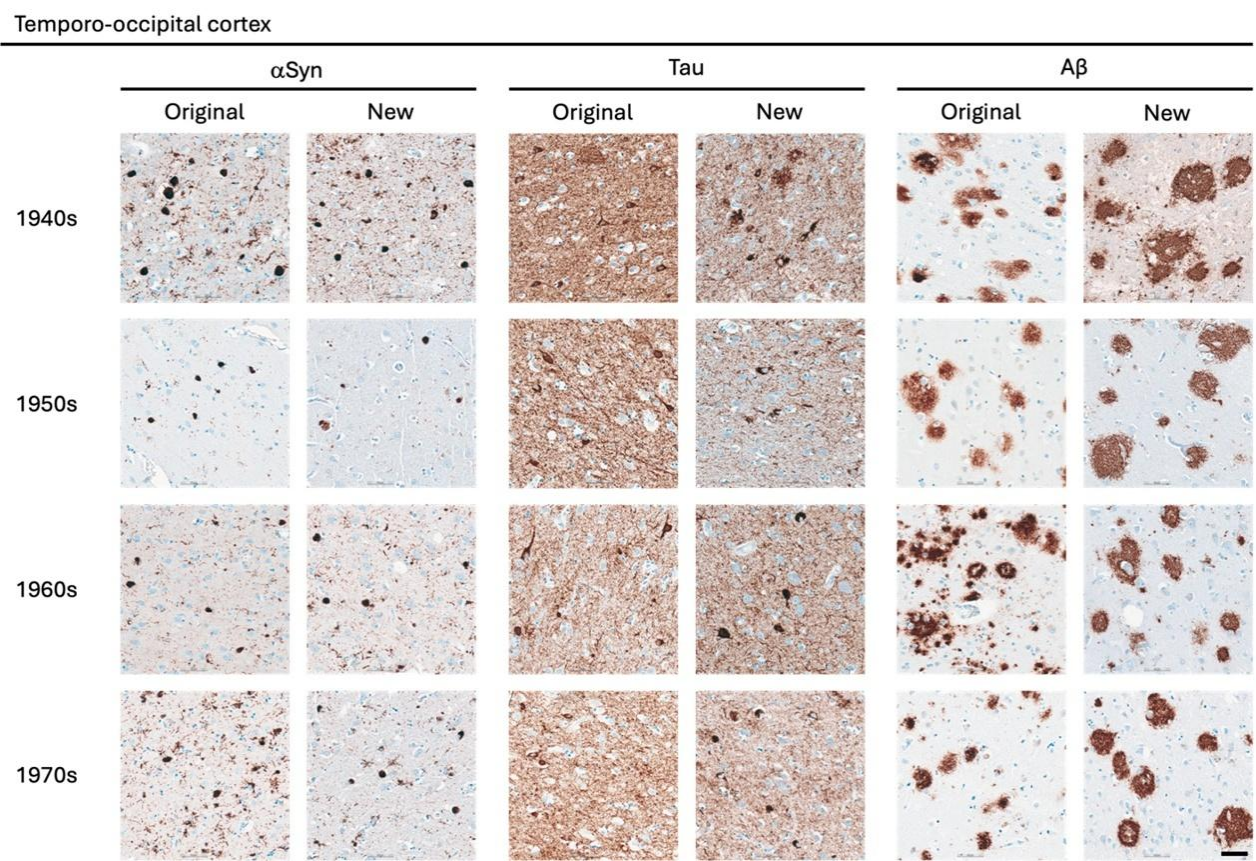
Representative images of immunohistochemical stains of temporo-occipital cortex for originally paraffin-embedded blocks (Original) and for newly paraffin-embedded blocks upon prolonged fixation (New). **αSyn** Photomicrographs comparing 5G4 IHC staining seen in same case per decade. **Tau** Photomicrographs comparing AT8 IHC staining seen in same case per decade. **Aβ** Photomicrographs comparing 4G8 IHC staining seen in same case per decade. Scale bar = 50 μm. Abbreviations: αSyn, alpha-synuclein; Aβ, amyloid-beta.

**Figure 4:**
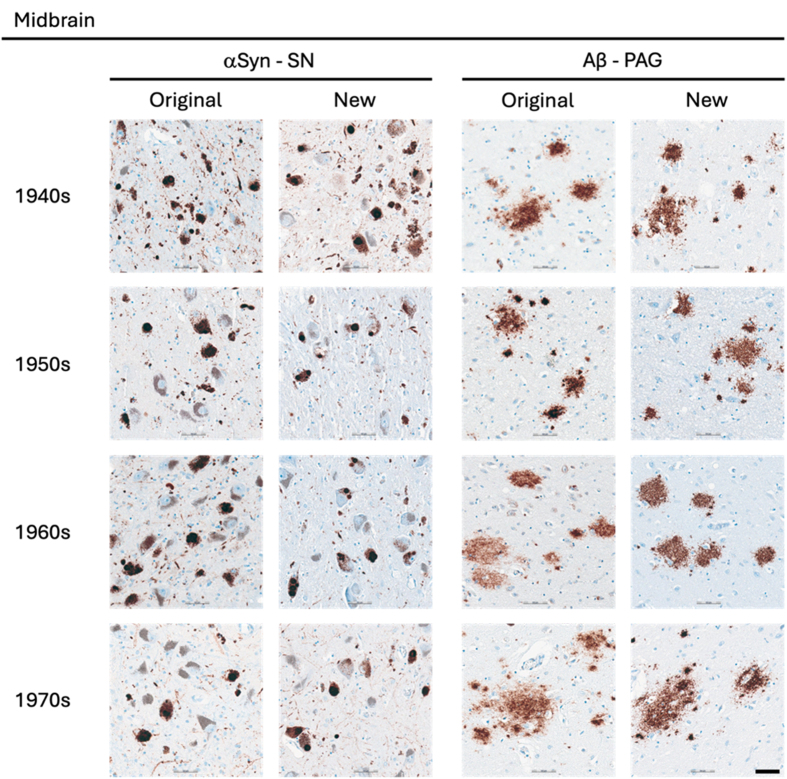
Representative images of immunohistochemical stains of midbrain for originally paraffin-embedded blocks (Original) and for newly paraffin-embedded blocks upon prolonged fixation (New). **αSyn** Lewy pathology labelled by 5G4 IHC in the SN across the four decades. **Aβ** Senile plaques detected with 4G8 IHC in original and new blocks across the decades. Scale bar = 50 μm. Abbreviations: αSyn, alpha-synuclein; Aβ, amyloid-beta; PAG, periaqueductal grey; SN, substantia nigra

**Figure 5:**
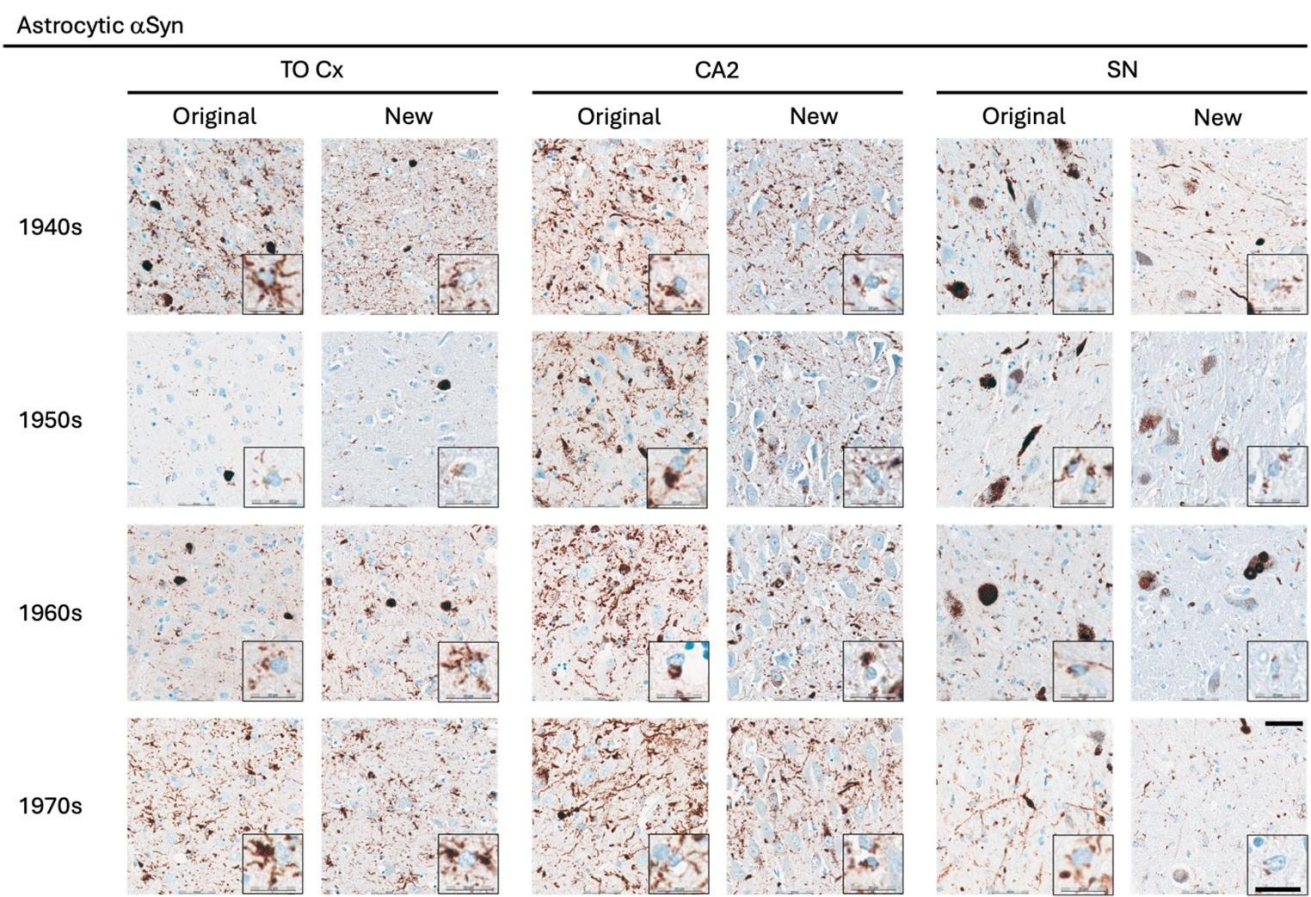
Representative images of immunohistochemical stains of astrocytic αSyn in temporo-occipital cortex, CA2 of the hippocampus and substantia nigra for originally paraffin-embedded blocks (Original) and for newly paraffin-embedded blocks upon prolonged fixation (New). Inserts show magnification of astrocytes containing αSyn labelled with the 5G4 antibody. Scale bars = 50 μm and 20 μm for inserts. Abbreviations: αSyn, alpha-synuclein; SN, substantia nigra; TO Cx, temporo-occipital cortex.

A total of 162 paraffin-embedded blocks were included, 394 IHC stains were performed and 944 regions were semi-quantitatively assessed. In general, good staining quality was achieved across all decades, ranging from 1946 to 1980, and across both types of tissue preservation, i.e. original blocks stored in cardboard boxes for up to 78 years and newly sampled blocks with fixation times up to 78 years. In the comparison below, the original blocks were regarded as the gold standard, and the antigenicity of new blocks was evaluated relative to that of original blocks. Representative images of the staining quality of αSyn, tau, and Aβ pathology across the decades are provided in **Fig. 1–5**.

### αSyn

The 5G4 antibody labelled both LBs and LNs with good staining quality and intensity in the original blocks. Comparable results were obtained in the new blocks with long fixation times (**Fig. 1–4**). Numerically, the new blocks showed slightly lower semi-quantitative scores than the original blocks, but no statistically significant differences were observed in any region (**Table 5**).

Astrocytic αSyn aggregates were generally adequately labelled (**Fig. 5**) and these also showed slightly lower numerical scores on average across most regions, but a statistically significant difference was seen only in the substantia nigra of the 1960s group (p < 0.05) (**Table 5**).

**Table 5:** Semiquantitative assessment of αSyn pathology

αSyn		Lewy Bodies			Lewy Neurites			Astrocytic Syn		
		Semiquantitative assessment			Semiquantitative assessment			Semiquantitative assessment		
	Sample size	Original Mean ± SEM	New Mean ± SEM	Wilcoxon test	Original Mean ± SEM	New Mean ± SEM	Wilcoxon test	Original Mean ± SEM	New Mean ± SEM	Wilcoxon test
**SN**										
1940s	n = 8	1.3 ± 0.5	0.9 ± 0.4	ns	1.4 ± 0.5	1.0 ± 0.4	ns	1.1 ± 0.5	0.8 ± 0.3	ns
1950s	n = 11	0.9 ± 0.4	0.8 ± 0.3	ns	0.9 ± 0.3	0.7 ± 0.3	ns	0.9 ± 0.3	0.6 ± 0.2	ns
1960s	n = 12	1.5 ± 0.4	1.0 ± 0.3	ns	1.4 ± 0.4	1.0 ± 0.3	ns	1.7 ± 0.4	1.1 ± 0.3	*
1970s	n = 8	2.4 ± 0.2	1.9 ± 0.1	ns	2.1 ± 0.1	1.9 ± 0.1	ns	2.1 ± 0.1	2.3 ± 0.2	ns
**CA2**										
1940s	n = 7	0.7 ± 0.4	0.6 ± 0.3	ns	1.0 ± 0.5	0.9 ± 0.4	ns	1.1 ± 0.6	1.1 ± 0.6	ns
1950s	n = 10	0.3 ± 0.2	0.2 ± 0.1	ns	0.7 ± 0.3	0.6 ± 0.2	ns	0.9 ± 0.4	0.8 ± 0.4	ns
1960s	n = 10	0.6 ± 0.2	0.3 ± 0.2	ns	1.1 ± 0.4	0.7 ± 0.2	ns	1.3 ± 0.4	0.9 ± 0.4	ns
1970s	n = 10	0.8 ± 0.3	0.9 ± 0.2	ns	2.0 ± 0.3	1.7 ± 0.2	ns	2.3 ± 0.3	2.1 ± 0.4	ns
**Ent Cx**										
1940s	n = 7	0.7 ± 0.4	0.9 ± 0.5	ns	0.7 ± 0.4	0.9 ± 0.5	ns	1.0 ± 0.5	1.0 ± 0.5	ns
1950s	n = 8	0.4 ± 0.3	0.4 ± 0.2	ns	0.8 ± 0.3	0.5 ± 0.2	ns	1.0 ± 0.4	0.5 ± 0.2	ns
1960s	n = 11	1.2 ± 0.3	0.9 ± 0.3	ns	1.2 ± 0.3	0.8 ± 0.2	ns	1.5 ± 0.4	1.2 ± 0.3	ns
1970s	n = 8	1.6 ± 0.2	1.5 ± 0.3	ns	1.8 ± 0.2	1.8 ± 0.3	ns	2.5 ± 0.3	2.3 ± 0.4	ns
**TO Cx**										
1940s	n = 6	1.0 ± 0.6	0.8 ± 0.5	ns	1.0 ± 0.6	0.8 ± 0.5	ns	1.0 ± 0.6	1.0 ± 0.6	ns
1950s	n = 9	0.8 ± 0.3	0.7 ± 0.3	ns	0.9 ± 0.3	0.7 ± 0.2	ns	1.0 ± 0.3	0.9 ± 0.4	ns
1960s	n = 11	1.1 ± 0.4	0.9 ± 0.3	ns	1.2 ± 0.4	0.8 ± 0.3	ns	1.3 ± 0.4	0.9 ± 0.4	ns
1970s	n = 10	2.3 ± 0.2	2.1 ± 0.3	ns	2.4 ± 0.2	1.9 ± 0.2	ns	2.7 ± 0.2	2.5 ± 0.2	ns

Abbreviations: **Ent Cx** - entorhinal cortex, **SN** - substantia nigra; **TO Cx** - temporo-occipital cortex. **ns** - non-significant; *, p < 0.05

### Tau

The AT8 antibody labelled NFTs and NTs with good staining quality and intensity in the original blocks across the decades (**Fig. 1–3**). In the 1940s group, the new blocks showed semi-quantitative scores comparable to the original blocks, with some slightly lower and some slightly higher, but no significant differences were observed in any region. The 1950s group displayed the greatest difference between sections from original and new blocks. Significant differences in semi-quantitative scores were found for NTs across all regions (CA2, p < 0.05; CA1, p < 0.01; Ent Cx, p < 0.05; TO Cx, p < 0.05), whereas for NFTs, significant differences were observed only in the CA2 region of the hippocampus (p < 0.05) and the TO Cx (p < 0.05). In the 1960s group, new blocks showed slightly lower semi-quantitative scores than original blocks, but no significant differences were detected for NFTs in any region. Labelling of NTs also showed slightly lower numerical scores on average, with significant differences detected only in the CA1 of the hippocampus (p < 0.05) and the Ent Cx (p < 0.05). In the 1970s group, new blocks again showed slightly lower semi-quantitative scores on average across most regions, with a significant difference seen only for NTs in the CA1 of the hippocampus (p < 0.05) (**Table 6**).

**Table 6:** Semiquantitative assessment of tau pathology

Tau		Neurofibrillary Tangles			Neuropil Threads		
		Semiquantitative assessment			Semiquantitative assessment		
	Sample size	Original Mean ± SEM	New Mean ± SEM	Wilcoxon test	Original Mean ± SEM	New Mean ± SEM	Wilcoxon test
**CA2**							
1940s	n = 7	2.4 ± 0.3	1.9 ± 0.4	ns	2.9 ± 0.1	2.4 ± 0.3	ns
1950s	n = 10	1.9 ± 0.4	0.9 ± 0.3	*	2.3 ± 0.3	1.1 ± 0.2	*
1960s	n = 10	1.3 ± 0.5	0.7 ± 0.3	ns	1.7 ± 0.4	1.3 ± 0.2	ns
1970s	n = 10	2.0 ± 0.3	1.3 ± 0.3	*	2.4 ± 0.3	1.8 ± 0.3	ns
**CA1**							
1940s	n = 8	2.4 ± 0.3	2.4 ± 0.4	ns	2.4 ± 0.3	2.3 ± 0.4	ns
1950s	n = 10	1.9 ± 0.3	1.2 ± 0.4	ns	2.1 ± 0.3	1.1 ± 0.3	**
1960s	n = 12	1.6 ± 0.4	1.3 ± 0.4	ns	1.8 ± 0.3	1.3 ± 0.2	*
1970s	n = 10	2.4 ± 0.2	2.0 ± 0.4	ns	2.3 ± 0.3	1.6 ± 0.2	*
**Ent** **Cx**							
1940s	n = 7	2.1 ± 0.5	2.1 ± 0.3	ns	2.3 ± 0.4	2.0 ± 0.3	ns
1950s	n = 8	1.9 ± 0.4	1.0 ± 0.4	ns	2.1 ± 0.4	1.0 ± 0.3	*
1960s	n = 11	1.9 ± 0.3	1.4 ± 0.4	ns	2.1 ± 0.3	1.5 ± 0.2	*
1970s	n = 8	2.3 ± 0.3	1.9 ± 0.4	ns	2.4 ± 0.3	1.9 ± 0.3	ns
**TO** **Cx**							
1940s	n = 6	2.3 ± 0.3	2.7 ± 0.2	ns	2.5 ± 0.3	2.8 ± 0.2	ns
1950s	n = 10	2.1 ± 0.3	1.2 ± 0.3	*	2.1 ± 0.3	1.2 ± 0.3	*
1960s	n = 11	1.5 ± 0.4	1.2 ± 0.4	ns	1.6 ± 0.3	1.3 ± 0.2	ns
1970s	n = 10	1.9 ± 0.3	1.8 ± 0.3	ns	2.2 ± 0.3	1.9 ± 0.3	ns

Abbreviations: **Ent Cx** - entorhinal cortex; **TO Cx** - temporo-occipital cortex. **ns** - non-significant; *, p < 0.05; **, p < 0.01

### Aβ

The 4G8 antibody reliably detected senile plaques in both types of preserved tissue, with good staining intensity and quality across all decades (**Fig. 1–4**). Comparisons between original and new blocks showed similar or slightly higher numerical scores in the new blocks across the decades and anatomical regions, but no significant differences were detected (**Table 7**). Cerebral amyloid angiopathy (CAA) was observed in only 6 of 41 cases, but in these cases, CAA was present in both cortical regions of the original and new blocks (data not shown).

**Table 7:** Semiquantitative assessment of amyloid- pathology

Aβ		Senile Plaques		
		Semiquantitative assessment		
	Sample size	Original Mean ± SEM	New Mean ± SEM	Wilcoxon test
**Ent** **Cx**				
1940s	n = 7	2.3 ± 0.5	2.4 ± 0.4	ns
1950s	n = 8	1.6 ± 0.5	2.3 ± 0.4	ns
1960s	n = 11	1.7 ± 0.5	1.8 ± 0.4	ns
1970s	n = 8	2.6 ± 0.4	2.6 ± 0.4	ns
**CA1**				
1940s	n = 8	1.3 ± 0.5	2.0 ± 0.4	ns
1950s	n = 10	1.4 ± 0.4	1.3 ± 0.4	ns
1960s	n = 12	1.0 ± 0.3	1.2 ± 0.4	ns
1970s	n = 10	1.9 ± 0.2	2.4 ± 0.3	ns
**CA2**				
1940s	n = 7	0.9 ± 0.3	0.9 ± 0.3	ns
1950s	n = 10	0.5 ± 0.3	0.5 ± 0.3	ns
1960s	n = 10	0.7 ± 0.3	0.7 ± 0.3	ns
1970s	n = 10	0.9 ± 0.4	0.5 ± 0.2	ns
**TO** **Cx**				
1940s	n = 6	2.7 ± 0.2	2.8 ± 0.2	ns
1950s	n = 9	2.7 ± 0.3	2.7 ± 0.3	ns
1960s	n = 11	1.8 ± 0.4	1.8 ± 0.4	ns
1970s	n = 10	2.7 ± 0.3	2.7 ± 0.3	ns
**PAG**				
1940s	n = 5	1.6 ± 0.5	2.0 ± 0.6	ns
1950s	n = 11	1.2 ± 0.4	1.2 ± 0.4	ns
1960s	n = 6	1.5 ± 0.5	1.7 ± 0.6	ns
1970s	n = 8	1.4 ± 0.3	1.8 ± 0.3	ns

Abbreviations: **Aβ** - amyloid-beta; **Ent Cx** - entorhinal cortex; **PAG** - periaqueductal grey; **TO Cx **- temporo-occipital cortex. **ns** - non-significant.

## Discussion

Some very old brain collections featured obsolete tissue handling, including formalin fixation protocols spanning years or decades, as in the present study. Yet, these collections represent a unique scientific resource, since the donors lived in earlier eras, prior to exposure to modern environmental toxicants and widespread use of contemporary medications. Here, we assessed αSyn, tau, and Aβ pathology in long-term stored paraffin-embedded blocks and in prolonged fixated tissue, with preservation times ranging from 45 to 78 years. To our knowledge, this is the first systematic investigation of the antigenicity of protein aggregates associated with AD and LBD in such old brain tissue. Our findings demonstrate that αSyn, tau, and Aβ aggregates can be satisfactorily detected using IHC when optimised antigen retrieval protocols are applied.

Detection of αSyn in long-fixated tissue has previously posed challenges. Pikkarainen and colleagues found that only clone 42, a commercial antibody against αSyn, detected αSyn-immunoreactive structures in long-fixed tissue [11]. Kovacs and colleagues introduced the 5G4 antibody, which labelled more neuritic and intracellular structures compared to clone 42 in archival tissue fixed up to 14 years. In that study, all antibodies except 5G4 showed background and/or normal synaptic (synaptophysin-like) staining, and 5G4 uniquely labelled astrocytic αSyn [14]. Altay and colleagues similarly reported that 5G4 and other αSyn antibodies targeting the central Non-Aβ Component of Alzheimer's disease amyloid (NAC) region of the αSyn, but not SYNO4, detected astrocytic αSyn [15]. Astrocytic αSyn accumulation, first described in the early 2000s [16], is increasingly recognised as a relevant component of LBD neuropathology [21]. Although regionally paralleling amygdala-predominant neuronal Lewy pathology, astrocytic aggregates differ biochemically, lacking ubiquitin and p62, and require NAC-targeting antibodies and FA pretreatment for detection [15,22,23]. Functionally, astrocytes may transition from protective clearance to pathogenic accumulation under lysosomal impairment [24]. Here, we demonstrate that astrocytic αSyn is detectable in paraffin-embedded tissue stored for up to 78 years, consistent with previous studies [14,15,23], further supporting its frequent association with neuronal Lewy pathology.

Given the broad range of the groups and fixation times, we examined only one antibody per protein aggregate. Further studies are needed to evaluate the efficacy of other antibodies targeting αSyn, Aβ, and hyperphosphorylated tau. Our aim was not to present specific protocol optimisations but to demonstrate the staining quality achievable with optimised protocols. Antigen retrieval using FA combined with heat was essential, particularly for αSyn and Aβ, consistent with prior studies [8,10,11,23,29]. Omission of FA markedly reduced αSyn antigenicity (data not shown). Based on previous studies, 5G4 was selected for αSyn detection, and our results confirm its utility even with fixation times up to 78 years. Similarly, the 4G8 antibody reliably detected senile plaques across antigen retrieval methods in archival brain tissue with prolonged fixation, consistent with earlier reports [10].

For tau, the AT8 antibody showed variable effects depending on the type of aggregate. Intracellular inclusions in long-fixed tissue maintained staining intensity and quality comparable to short-fixed tissue, whereas NTs showed reduced labelling. This aligns with previous observations of limited changes in NFT numbers despite prolonged fixation [9,11]. Earlier work by Dwork and colleagues reported good labelling of the Alz 50 epitope after 10 years of fixation, but reduced immunoreactivity with longer fixation times, and contrary to our findings, the immunosignal was completely absent after 30 years of fixation [30]. This discrepancy underscores that antibody selection and protocol optimisation critically influence detection of protein aggregates in long-fixed tissue [11].

This study has several limitations. First, sampling of new blocks depended on the original neuropathological sampling (1946–1980), and in some cases, exact adjacent sampling was not feasible. Thus, biological variation may have influenced results, as reduced pathology in new blocks could reflect regional differences rather than antigen loss. Second, pathological burden for each case was unknown prior to inclusion; some groups were positive for all three pathologies, whereas others comprised mixed negative and positive cases. Higher semi-quantitative scores for LBs and LNs in the 1970s group likely reflect group composition rather than preservation effects. Sample sizes, particularly for the oldest groups regarding αSyn, were limited. Approximately half of the cases initially identified as potential αSyn-positive were negative upon screening in the SN, limiting inclusion. Third, we observed a lower frequency of CAA than expected based on the burden of SPs. This may be explained by the limited cortical representation, as only entorhinal and temporo-occipital cortices were available, while frontal and occipital cortices were not consistently represented among the original blocks.

Across pathologies, a slight reduction in antigenicity was observed in new blocks compared to original blocks. However, differences between preservation categories were smallest in the 1940s and 1970s groups. This suggests that antigenicity loss may plateau after several years of fixation, with additional decades not proportionally exacerbating antigen degradation. Antigen visualisation depends on both antibody characteristics and pretreatment methods, as widely reported [8,10,11,14,15,23,31]. Consequently, investigating antigen decay in very long-fixed tissue is challenging, since negative IHC results may reflect a suboptimal antibody or antigen retrieval protocol rather than true antigen loss. Limited documentation of fixative type, postmortem delay, and fixation duration for the original blocks precluded assessment of their individual contributions. Nevertheless, as same-case comparisons were performed, the impact of these variables was likely minor.

Instances of apparent "false-negative" and "false-positive" IHC staining were observed when comparing new and original blocks (see coloured sections of **Table 4**). Discrepant results may reflect technical variability; however, the use of an automated immunostainer minimised procedural variation and improved reproducibility. Alternatively, minor anatomical differences between sampled regions may explain discordant findings, with negative staining in one block and positive staining in another reflecting true pathological distribution. In most discrepant cases, only one antibody in a single region was affected, while other regions and antibodies demonstrated adequate-to-good immunoreactivity.

Postmortem delay represents another factor influencing antigenicity. One case showed discrepant tau labelling across all regions, whereas αSyn pathology was consistently well-labelled in both preservation types. However, the postmortem delay in this formalin-fixed case was 25 hours, which is within commonly accepted limits. Together with the semi-quantitative analysis, this could indicate that tau antigenicity may be particularly vulnerable to prolonged fixation and potentially other factors, complicating epitope unmasking and antigen retrieval. Evaluation of other pre-sectioning factors was beyond the scope of this study, as the necessary information to explore these parameters were unavailable to us.

Overall, our findings indicate that protein aggregates characteristic of AD and LBD can be detected with adequate-to-good IHC quality in most archival cases, despite postmortem delay ranging from 6 to 113 hours, storage times up to 78 years, and fixation times up to 78 years. Future investigations should evaluate panels of antibodies against αSyn, tau, and Aβ to define the range and limitations of available antibodies in archival brain tissue studies. Exploration of neuroinflammatory markers and astrocytic αSyn using antibodies against truncated αSyn species may further elucidate pathology in long-fixed tissue. Furthermore, application of tissue clearing, spatial transcriptomics, and assessment of DNA and RNA integrity in both types of archival tissue should also be investigated.

In conclusion, with appropriate antigen retrieval methods and antibody selection, archival brain tissue stored either as paraffin-embedded blocks or in fixative for up to 78 years remains suitable for evaluation of defining neuropathological features of LBD and AD.

## Conflict of interest statement

The authors declare no conflict of interest.
